# Biting the Finger-nails

**Published:** 1892-04

**Authors:** 


### BITING THE FINGER-NAILS.

"It makes me positively nervous to see people bite their finger-nails," said a woman physician, as she stepped out of a street car with her friend. Positively nervous; and I sometimes feel as though I would like to rise to my feet in street car or stage, or wherever I happen to be, and then and there deliver a lecture on this dangerous practice. I believe that scores of people die every year from the effects of biting their nails. You might wonder how that can be, but you would not if you had been present at a post-mortem, as I was once, and seen the entire coating of the stomach bristling with bits of finger-nails. It was a sight that I shall never forget, and, when I see people indulging in this pernicious practice, I feel like warning them against such a dangerous habit. Aside from the physiological phase of this subject, it is any thing but pleasant to see persons gnawing at the ends of their fingers.

It is said that this habit indicates a bad disposition; but my observation leads me to believe that it is quite as likely to accompany nervous conditions as any defect in the temper. The habit is comparatively easy to break off if one goes at it in the right way. A middle-aged patient of mine, after having bitten her nails almost her entire lifetime broke herself of the habit by beginning on one finger. This she persistently left alone and carefully cultivated the finger nail, giving a certain amount of attention to it every day. When this finger-nail had grown to the usual length she took up another, and so on, until all her nails except one were in perfect shape. It took months of the most persistent effort to break up the last remaining scrap of this tenacious practice. She said that she thought it was actually the greatest struggle of her life. The habit had become so fixed, that after days of abstinence from even a single nibble she would find herself almost viciously gnawing away at the poor little finger, but after awhile the effort was a success, and although her hands never regained their symmetrical shape, they were vastly improved, and her health which had been seriously affected, improved also. She had for years been subject to indigestion and kindred difficulties, but, for some reason or other, they almost entirely left her. She could not be persuaded to believe that the breaking up of the habit of biting her finger-nails had any thing to do with her improved health, but I always entertained a very decided opinion on the subject.

